# Interactions between cyclodextrins and cellular components: Towards greener medical applications?

**DOI:** 10.3762/bjoc.12.261

**Published:** 2016-12-07

**Authors:** Loïc Leclercq

**Affiliations:** 1 Univ. Lille, CNRS, ENSCL, UMR 8181 – UCCS - Equipe CÏSCO, F-59000 Lille, France

**Keywords:** cellular interactions, cyclodextrins, endogenous substances, extraction, greener active ingredients, host–guest chemistry, lipids

## Abstract

In the field of host–guest chemistry, some of the most widely used hosts are probably cyclodextrins (CDs). As CDs are able to increase the water solubility of numerous drugs by inclusion into their hydrophobic cavity, they have been widespread used to develop numerous pharmaceutical formulations. Nevertheless, CDs are also able to interact with endogenous substances that originate from an organism, tissue or cell. These interactions can be useful for a vast array of topics including cholesterol manipulation, treatment of Alzheimer’s disease, control of pathogens, etc. In addition, the use of natural CDs offers the great advantage of avoiding or reducing the use of common petroleum-sourced drugs. In this paper, the general features and applications of CDs have been reviewed as well as their interactions with isolated biomolecules leading to the formation of inclusion or exclusion complexes. Finally, some potential medical applications are highlighted throughout several examples.

## Introduction

Cyclodextrins (CDs) were discovered and identified over a century ago [1–3]. Between 1911 and 1935, Pringsheim and co-workers demonstrated the ability of CDs to form complexes with many organic molecules [4–5]. Since the 1970s, the structural elucidation of the three natural CDs, α-, β-, and γ-CDs composed of 6-, 7-, and 8-membered α-D-glucopyranoses linked by α-1,4 glycosidic bonds, allowed the development and the rational study of their encapsulation properties [6–7]. As their water solubility differs significantly, a great variety of modified CDs has been developed to improve the stability and the solubility of inclusion complexes [8–10]. Nowadays, CDs are widely applied in many fields [11–28] due to their host–guest properties, their origins (produced from starch by enzymatic conversion), their relatively low prices, their easy modifications, their biodegradability and their low toxicity. Moreover, CDs are able to interact with a wide range of biomolecules opening the way for many biological applications. The majority of these researches are based on the ability of CDs to extract lipids from the cell membrane. The objective of this contribution is to focus on the potential use of natural and chemically modified CDs in the vast array of medical and biological applications.

## Review

### Cyclodextrins: synthesis, structure and physicochemical properties.

#### i) Native cyclodextrins

**Scheme 1:**
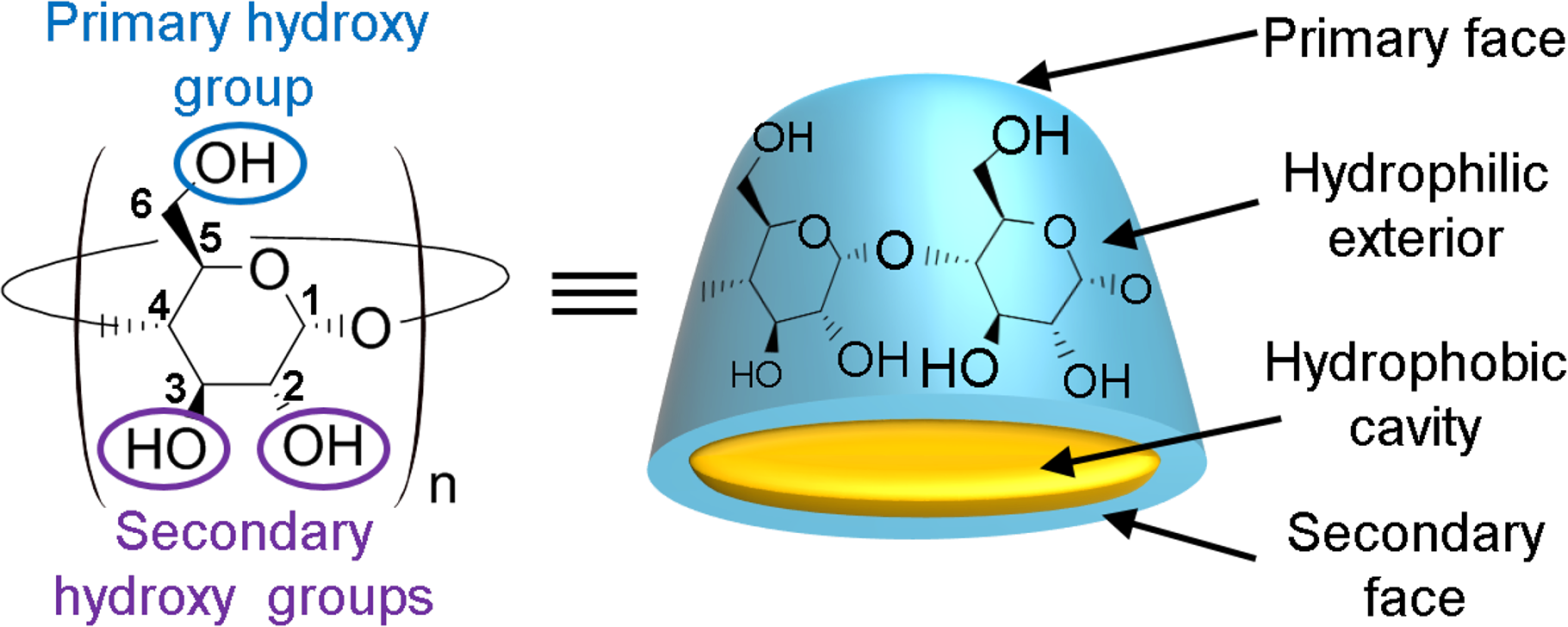
Structure and conventional representation of native CDs.

As mentioned earlier, the ordinary starch hydrolysis (e.g., corn starch) by an enzyme (i.e., cyclodextrin glycosyl transferase, CGTase) allows the production of the native CDs [13]. To reduce the separation and the purification costs, selective α-, β- and γ-CGTases have been developed in the last two decades [29]. Nevertheless, the cheapest remains the β-CD whereas the most expensive is the γ-CD. The molecular shape of the native CDs can be represented as a truncated cone with “hydrophobic” cavity which can accommodate hydrophobic compounds (Scheme 1). In aqueous solution, the complexation is enthalpically and entropically driven. In addition, complementary interactions (e.g., van der Waals forces, H-bonds, etc.) appear between the CD and the guest. The non-polar suitably-sized guest may be bound in numerous molar ratios (e.g., 1:1, 2:1, 1:2, etc.). In all cases, the knowledge of the binding constants (*K*_ass_) is crucial because these values provide an index of host–guest binding forces. CDs can also form exclusion complexes where the CDs are bound to the guest through a H-bond network. For instance, the complexation of [PMo_12_O_40_] anion by β- and γ-CD results in a one-dimensional columnar structure through a combination of intermolecular [C−H···O=Mo] and [O−H···O] interactions [30]. Unfortunately, the natural CDs as well as their inclusion complexes are of limited aqueous solubility leading to their precipitation. Fortunately, native CDs are effective templates for the generation of a wide range of molecular hosts through chemical modifications.

#### ii) Modified cyclodextrins

In order to meet specific requirements in the host–guest complex, chemical modifications make it possible to tailor CDs to a particular guest. The hydroxy groups serve as scaffolds on which substituents can easily be introduced. From a chemical synthesis point of view, the reactivity difference between the primary and secondary hydroxy groups allows selective functionalization on the narrow or the wider edge of the truncated cone (Table 1). Access to the gamut of functional groups greatly expands the utility of native and modified CDs in their numerous applications.

**Table 1:** Structures, acronyms and characteristics of some modified cyclodextrins.^a^

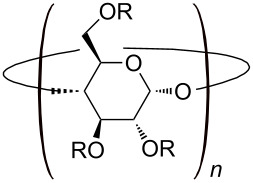		

Abbreviation	Substituents (R)	Characteristics

ME	–H or –CH_3_	soluble in cold water and organic solvents, hemolytic
HP	–H or –CH_2_CH(OH)CH_3_	highly water-soluble, low toxicity
S	–H or -SO_3_Na	p*K*_a_ > 1, water soluble
SBE	–H or –(CH_2_)_4_SO_3_H	water soluble
G1	–H or –glucosyl	highly water soluble
G2	–H or –maltosyl	low toxicity

^a^ME: methyl; HP: 2-hydroxypropyl; S: sulfate; SBE: sulfobutyl ether; G1 glucosyl; G2: maltosyl.

#### iii) Applications of cyclodextrins

As natural CDs and their derivatives are able to encapsulate a wide range of guest molecules into their cavity, they can be used in a wide range of applications including analytical chemistry [21–22,31], agriculture [15], food technology [16], catalysis [23–25,32], cosmetics [26], textile processing [28,33], and environmental protection technologies [27,34]. Nevertheless, the first global consumer of CDs is clearly the pharmaceutical industry [35–36]. Indeed, CDs are very useful to form inclusion complexes with a wide range of drugs and become a very valuable tool for the formulator in order to overcome delivery limitations [37–38]. As a result, numerous formulations that use CDs are now on the market worldwide (Table 2).

**Table 2:** Some marketed pharmaceutical formulations with CD.^a^

CD	Drug	Formulation	Trade name	Market

α-CD	Alprostadil	IV solution	Rigidur	Europe, USA
α-CD	Cefotiam hexetil	Oral tablet	Pansporin T	Japan
β-CD	Iodine	Topical solution	Mena-Gargle	Japan
β-CD	Nicotine	Sublingual tablet	Nicorette	Europe
β-CD	Piroxicam	Oral tablet	Flogene	Brazil, Europe
HP-β-CD	Hydrocortisone	Topical cream	Dexocort	Europe
HP-β-CD	Itraconazole	IV solution	Sporanox	Europe, USA
HP-β-CD	Mitomycin	IV solution	Mitozytrex	USA
ME-β-CD	Chloramphenicol	ED solution	Clorocil	Europe
SBE-β-CD	Voriconazole	IV solution	Vfend	Europe, USA
SBE-β-CD	Ziprasidone	IM solution	Zeldox	Canada, USA
γ-CD	Minoxidil	Topical solution	Alopexy	Europe
HP-γ-CD	Diclofenac	ED solution	Voltarenopthta	Europe

^a^Note that the list is not exhaustive and that only one trade name is given (IV: intravenous, IM: intramuscular, ED: eye drop). Adapted from [37].

#### iv) Toxicity and biological effects of native and modified cyclodextrins

As safety and toxicity are important criteria for consideration before using CDs in pharmaceutical products, this section deals with toxicological issues. The native α- and β-CD, unlike γ-CD, cannot be hydrolyzed by pancreatic amylases and human salivary but can be fermented by the intestinal microflora. When administered orally, native CDs and hydrophilic derivatives are not absorbed from the human gastrointestinal tract and thus making them practically nontoxic due to their high molecular mass ranging from almost 1 000 to over 2 000 g/mol and their hydrophilic nature with a significant number of H-bond donors and acceptors [39]. Indeed, CDs violate three criteria of the Lipinski’s rule: i) no more than 5 H-bond donors, ii) no more than 10 H-bond acceptors, iii) a molecular mass less than 500 g/mol, and iv) an octanol–water partition coefficient (log *P*) not greater than 5 [40]. As these criteria apply only to absorption by passive diffusion of compounds through cell membranes, the absorption of the native CDs and their hydrophilic derivatives are not allowed in their intact form and any cellular absorption, if it occurs, is by passive transport through cytoplasmic membranes (i.e., by transporter proteins) [41]. In contrast, lipophilic derivatives (e.g., ME-β-CD) interact more readily with membranes than the hydrophilic derivatives, they cannot readily permeate cell membranes (see below) [42]. Moreover, oral administration of alkylated CD derivatives, such as ME-β-CD, is limited by its potential toxicity [43]. Indeed, ME-β-CD is partially absorbed from the gastrointestinal tract into the systemic circulation. Moreover, they have been shown to be toxic after parenteral administration. The opposite holds for hydrophilic CD derivatives, such as HP-β-CD and SBE-β-CD, which are considered safe for parenteral administration. In a general way, the γ-CD, HP-β-CD and SBE-β-CD, S-β-CD and G2-β-CD appear to be globally safer than α-, β- and alkylated CDs which are less suitable for parental administration [44–45]. Table 3 presents the pharmacokinetics and safety overview of some natural and modified CDs. When administered, natural and hydrophilic CD derivatives disappear rapidly from systemic circulation and are distributed to various tissues of the body such as kidney, liver, urinary bladder, etc. Nevertheless, they are mainly renally excreted intact. At high concentrations, α-, β- and alkylated CDs present renal damage and dysfunction [46]. In 2008, Stella and He discussed the detailed studies of toxicology, mutagenicity, teratogenicity and carcinogenicity of various CDs [45]. Overt signs of acute toxicity are not apparent for CDs (i.e., no inflammatory response and no cell degeneration). They are also not genotoxic, not teratogenic or mutagenic. However, CDs affect the human organism only at extremely high concentrations. Nevertheless, the principal side effect of natural and modified CDs is probably the cell toxicity. This effect is directly correlated to their hemolytic activities. Indeed, several in vitro studies reported erythrocyte lysis although the toxicological implication in vivo is negligible. The lysis mechanism is related to their capacity to draw phospholipids and cholesterol out of the biological membrane (see below). Based on this, the complexation of endogenous substances are of potential interest for many applications.

**Table 3:** Pharmacokinetics and safety overview of some native and modified CDs for rats.^a^

CD	Fraction excreted unchanged in urine	Oral adsorption	LD_50_ oral (g/kg)	LD_50_ IV (g/kg)

α-CD	≈90%	2–3%	>10	0.5–0.75
β-CD	≈90%	1–2%	>5	0.45–0.79
γ-CD	≈90%	<0.02%	>>8	4
HP-β-CD	≈90%	≤3%	>2	10
G_2_-β-CD	–	–	>5	–
ME-β-CD	>95%	0.5–12%	>8	1.5–2.1
SBE-β-CD	–	–	>10	>15

^a^Taken from [44,47–53]. ^b^ Randomly methylated β-CD.

### Biomolecule/cyclodextrin inclusions complexes

Native and modified CDs can be used to complex certain chemicals produced naturally present in cells and tissues (i.e., endogenous substances). Indeed, CDs are able to form complexes with various biomolecules including lipids, carbohydrates, proteins and nucleic acids. In this section, some biomolecule/CD inclusion complexes are presented.

#### i) Complexation of lipids and consequences

Lipids are hydrophobic or amphiphilic molecules very diverse, including, among other fats, waxes, sterols, fat-soluble vitamins, phospholipids, mono-, di- and triglycerides, etc. Their amphiphilic nature causes the molecules of certain lipids to organize into liposomes when they are in aqueous medium. This property allows the formation of biological membranes. Indeed, cells and organelles membranes are composed of lipids. Lipids also provide various other biological functions, including cell signaling and storage of metabolic energy by lipogenesis. Biological lipids are basically due to two types of compounds acting as “building blocks”: ketoacyl groups and isoprene units. From this point of view, they can be divided into eight categories: fatty acids (and their derivatives: mono-, di- and triglycerides and phospholipids), acylglycerols, phosphoglycerides, sphingolipids, glycolipids and polyketides, which result from the condensation of ketoacyl groups, sterols (e.g., cholesterol) and prenols, which are produced from condensation of isoprene units [54]. These compounds can be easily included inside the CDs because they are hydrophobic or amphiphilic molecules. As mentioned earlier, and as it will become exceeding clear throughout the following sections, the majority of research involving CDs has revolved around their ability to manipulate lipid (phospholipids and cholesterol) composition in different cells [55–58]. Although numerous studies deal of this topic, the mechanism of this process is poorly investigated (i.e., only the consequences of this phenomenon are reported). For sake of clarity, only some typical examples are reported in this section.

The first well-documented effect of CDs is probably hemolysis which corresponds to the lysis of red blood cells (erythrocytes) and the release of their contents into surrounding fluid (blood plasma). In 1982, Irie and co-workers reported that native CDs are able to cause hemolysis of human erythrocytes [59]. This behavior occurs at relatively high concentrations (>1 mM) and that the degree of cholesterol extraction is a function of the CD used, its concentration, incubation time, temperature. For instance, in given conditions (isotonic solution with similar incubation time and temperature), the observed hemolysis is in the order γ-CD < α-CD < β-CD.

This different effect, observed for native CDs, has been explained by Ohtani et al. in 1989 [58]. As the membrane of erythrocytes is composed of proteins (43%) associated with lipids (49%) and carbohydrates (8%) and as the fraction of cholesterol is 25% of total membrane lipids [54], the proposed explanation is based on the specific interaction of natural CDs with the erythrocyte membrane components. Indeed, α- and β-CD are excellently suited to solubilize phospholipids and cholesterol, respectively, whereas γ-CD is generally less lipid-selective. In more detail, the CD affinity for solubilizing various lipid components of the erythrocyte membranes are in the order γ-CD << β-CD < α-CD for phospholipids and α-CD < γ-CD << β-CD for cholesterol [58]. These findings are corroborated by the work of Leventis and Silvius which have reported that β- and γ-CD accelerate the rate of cholesterol transfer by a larger factor than they accelerate the transfer of phospholipid, whereas the opposite is true for α-CD [60]. The hemolytic properties of CDs are a general behavior not limited to human erythrocytes: All mammalian red blood cells are affected by the parent CDs. For instance, dog erythrocytes are also affected by native CDs in the order γ-CD < α-CD < β-CD [61]. Thus, the magnitude of the hemolytic activity observed for dog erythrocytes is consistent with the order of magnitude of human erythrocytes (see discussion above). However, the hemolytic activity is largely influenced by the substituents attached to the CDs. The presence of hydrophilic substituents (e.g., glucosyl, 2-hydroxypropyl, 3-hydroxypropyl, maltosyl, sulfate, sulfobutyl ether, etc.) reduces the hemolytic activity in comparison with the parent CDs while the lipophilic ones (e.g., methylated CDs) demonstrate the strongest hemolytic activities [62]. As for parent CDs, these differences are ascribed to the different solubilization effects of lipid components and their sequestration in the external aqueous phase.

As the hemolysis is attributed to the removal of erythrocyte membrane components, particularly phospholipids and cholesterol, the value of the binding constants between CDs and lipids can be very relevant. To the author’s knowledge there is only one paper in the literature that describes the binding constants between CDs (α- or γ-CD) and short phospholipids (i.e., diheptanoylphosphatidylcholine, DHPC) [63]. In this study, the association constants were estimated from ^1^H NMR measurements. The results proved that the *K*_1_ values are in the order α-CD < γ-CD while the *K*_2_ values are in the order γ-CD < α-CD. This behavior was attributed to the large cavity of γ-CD which is able to incorporate both alkyl chains of DHPC simultaneously. In contrast, the formation of a 1:2 inclusion complex with α-CD is easier than with γ-CD. These findings are corroborated by Fauvelle and co-workers who reported that α-CD has the strongest affinity to phospholipids (e.g., phosphatidylinositol) [64–65]. In 2000, Nishijo et al. studied the interactions of various CDs with dipalmitoyl, distearoyl, and dimyristoylphosphatidylcholine liposomes. This study highlights that the liposome-CD interaction depends on the length of the fatty acid chain of the phospholipid, the cavity size, and the nature of the substituents at the CD [66]. In the literature, the binding constant between cholesterol and β-CD was estimated around 1.7 × 10^4^ M^−1^ from a solubility method [67–68]. This value proves the good stability of the inclusion complex because of the driving force of complexation: hydrophobic interaction. Despite there is no information on the binding constant observed for the inclusion of cholesterol in γ-CD, the cavity internal diameter of the β-CD and its derivatives perfectly matches the size of the sterol molecules contrary to γ-CD (too large) [69–70]. Moreover, the positive correlation observed between the hemolytic activities of various modified CDs and their ability to solubilize cholesterol reveal that HP-β-CD was shown to be a more efficient cholesterol-acceptor molecule than HP-γ-CD. This is apparently due to the diameter of its internal cavity that matches the size of this molecule. Finally, it is noteworthy that all CDs lose their abilities to induce hemolysis, when their cavities are occupied with guest molecules due to a reduced interaction with the erythrocyte membranes [71]. All these observations support the aforementioned affinities of α-CD for phospholipids and of β-CD for cholesterol.

**Scheme 2:**
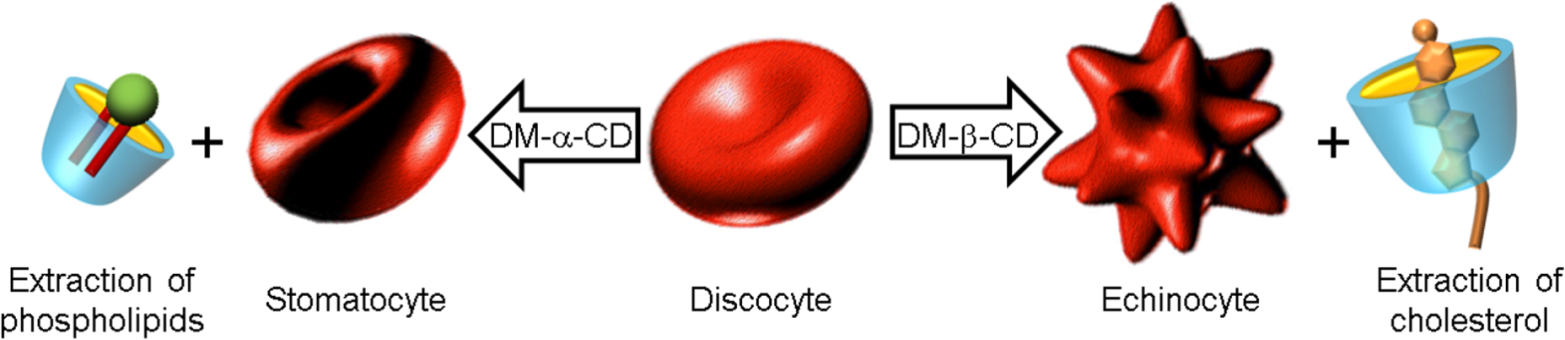
Proposed mechanism for morphological changes in erythrocytes induced by methylated CDs.

In addition, sub-hemolytic concentrations of native CDs are also demonstrated to cause shape changes in human erythrocytes. The hemolytic effect is concomitant with shape changes (from biconcave discocyte to stomatocyte or echinocyte) depending on the cavity size of the CDs. For instance, α- and γ-CD induce progressive shape changes from discocytes into stomatocytes and from stomatocytes into spherocytes [58]. In contrast, β-CD leads only to swelling of erythrocytes. Similar effects are found for chemically modified CDs. For instance, Motoyama et al. have reported morphological changes in erythrocytes induced by methylated CDs such as 2,6-di-*O*-methyl-α-CD and 2,6-di-*O*-methyl-β-CD (DM-α-CD and DM-β-CD) [72–73]. The authors reported that DM-α-CD induces morphological changes in rabbit erythrocytes leading to stomatocytes, while DM-β-CD leads to echinocytes. This difference is ascribed to the cavity size of the CDs and their ability to extract either sphingomyelin and/or cholesterol of lipid rafts. Lipid rafts are constituted of cholesterol, glycolipids, and sphingomyelin. Nevertheless, their structures have heterogeneity with the presence of cholesterol-rich and sphingomyelin-rich domains. Consequently, DM-α-CD extract sphingomyelin form sphingomyelin-rich domains while DM-β-CD extract cholesterol from cholesterol-rich lipid rafts (Scheme 2). This assertion is supported by the work of Nishijo et al. which reported the binding constants of the 1:1 and 1:2 (cholesterol:DM-β-CD) complexes (1.09 × 10^2^ M^−1^ and 5.68 × 10^4^ M^−1^, respectively, at 25 °C) [74]. Finally, it is noteworthy that the presence of DM-α-CD and DM-β-CD also leads to hemolysis. As cholesterol interacts with markedly higher affinity with sphingolipids (e.g., sphingomyelin) than with common membrane phospholipids, the extraction of cholesterol by DM-β-CD or sphingomyelin by DM-α-CD leads to strong modification of the cholesterol-rich or sphingomyelin-rich lipid rafts, respectively [60]. Therefore, even if the target is significantly different, the final effect is the same (i.e., hemolysis).

It should be noted that this cholesterol and/or phospholipids extraction is not limited to erythrocytes. Indeed, all eukaryotic or prokaryotic cells are affected by the presence of CDs. For instance, the cytotoxicity of native, methylated, and hydroxypropylated α-, β-, and γ-CDs have been studied on an in vitro model of blood-brain barrier by Monnaert and co-workers [75]. The results prove that the native CDs are the most toxic (γ-CD < β-CD < α-CD). As expected, lipid effluxes on the brain capillary endothelial cells in the presence of native CDs reveal that α-CD extracts only phospholipids whereas β-CD is able to remove phospholipids and cholesterol. In contrast, γ-CD is less lipid-selective than the other native CDs. This differential effect compared to the order of magnitude of hemolytic activity (γ-CD < α-CD < β-CD) could be ascribed to the lower cholesterol content in blood–brain barrier cells compared to erythrocytes. Indeed, the cholesterol fraction is markedly higher in erythrocytes than in other cells. As for hemolysis, the presence of hydrophilic substituents (e.g., 2-hydroxypropyl and sulfobutyl ether) annihilates the cytotoxicity while the presence of methyl residues induces cell death of various cells (Caco-2, TR146, PC-12, etc.) [76–78]. For instance, cell death induced by DM-β-CD is caused by a marked apoptosis mechanism (i.e., a process by which cells trigger their self-destruction in response to a signal which leads to cell changes prior to death) for NR8383, A549 and Jurkat cells [79]. This apoptosis process results from cholesterol extraction leading to inhibition of the activation of PI3K-Akt-Bad pathway. The presence of DM-α-CD had repercussions that were totally opposite to the DM-β-CD. Indeed, the cell death results from a non-apoptotic mechanism (i.e., necrosis). This differential effect could be attributed to a dissimilarity of interaction between the methylated CDs with the cholesterol-rich lipid rafts and with the sphingomyelin-rich domains for DM-β-CD and DM-α-CD, respectively. These results suggest that lipid rafts of cell membranes would be involved in cell death and cellular function.

However, the mechanism of cholesterol extraction mediated by CDs is still open to discussion. For instance, Yancey et al. proposed that CDs diffuse into the proximity of the erythrocyte membrane leading to lipid complexation without complete desorption in the aqueous phase [80]. In contrast, Besenicar et al. proposed that CDs complex lipids during their naturally exchange from the membrane to the aqueous phase [81]. Stella and He supposed that the CDs interact directly with the membrane of the cells prior to lipid efflux [45]. In the same idea, Mascetti et al. proposed that CDs interact directly with cholesterol [82]. Based on molecular simulations, López et al. proposed the following mechanism: i) association of CDs in aqueous solution to form dimers, ii) binding of dimers at the membrane surface, iii) extraction and complexation of cholesterol, and iv) desorption of CD/cholesterol in the aqueous solution [83]. However, whatever the molecular mechanism, the lipid efflux mediated by CDs is clearly different from those of surfactants. Indeed, at low concentrations, the mechanism involves the penetration of the detergent molecules into the lipid membrane leading to increase its fluidity. In contrast, at higher concentrations, the extraction of membrane constituents is ensured by micellar solubilization [84–85].

#### ii) Complexation of peptides and proteins and some applications

Proteins are polymers of amino acids covalently linked through peptide bonds. The nature of the proteins is determined primarily by their amino acid sequence, which constitutes their primary structure. Amino acids have very different chemical properties; their arrangement along the polypeptide chain determines their spatial arrangement. This is described locally by their secondary and tertiary structures. The secondary structure describes the arrangement of amino acid residues observed at the atomic scale stabilized mainly by H-bonds (e.g., α-helix, β-sheet and turns). The tertiary structure corresponds to the general shape of the observable protein across the whole molecule. It describes the interactions between the different elements of the secondary structure. Finally, the assembly of several protein subunits to form a functional complex is described by the quaternary structure [54]. As some amino acids have hydrophobic side chains (e.g., alanine, valine, leucine, isoleucine, proline, phenylalanine, tryptophan, cysteine and methionine), they can be easily included inside the CDs. This complexation leads to modification of the protein. For sake of clarity, only some typical examples are reported in this section.

In their paper on the differential effects of natives CDs, Ohtani and co-workers highlighted that, in addition to the extraction of lipids, these CDs are also able to solubilize proteins in the order α-CD < γ-CD << β-CD [58]. In 1991, Sharma and Janis studied the interaction of CDs with hydrophobic proteins leading to the formation of soluble and insoluble complexes [86]. CDs caused the precipitation of lipoproteins in the order γ-CD < α-CD < β-CD. This behavior could be ascribed to the formation of inclusion complexes. However, the presented data did not exclude the formation of exclusion complexes.

Several years later, Horský and Pitha reported a study on the interaction of CDs with peptides containing aromatic amino acids [87]. From competitive spectrophotometry measurements, with *p*-nitrophenol as a competing reagent at pH 7.4, the authors determined the stability constants of aromatic amino acids and their oligopeptides with α-, β-, HP-β-, and ME-β-CD. The estimated constants of free L-phenylalanine (Phe) increased in the order ME-β-CD ≈ HP-β-CD < α-CD < β-CD. Moreover, the results proved that the stability of oligopeptides containing Phe is higher than that of Phe itself. For instance, the binding constant of free Phe with β-CD was estimated at 17 M^−1^ whereas with the Gly-Gly-Phe tripeptide, the binding constant was 89 M^−1^. Nevertheless, the complexation occurs when the native functional form of the proteins is unfolded.

In 1996, Bekos et al. investigated the role of the L-tyrosine (Tyr) residue in the binding of pentapeptides to α- and β-CD [88]. The two peptides used in this study were: Tyr-Ile-Gly-Ser-Arg (YIGSR) and Tyr-Gly-Gly-Phe-Leu (YGGFL). The former interacts specifically with the integrin receptors on specific neuronal cells whereas the latter is known to bind to brain receptor sites. From steady-state fluorescence spectroscopy, the estimated constants of free Tyr increased in the order α-CD < β-CD. As in the previous study, the stability constants of pentapeptides containing Tyr with β-CD were higher than that of Tyr itself (48, 224, and 123 M^–1^ for free Try, YIGSR, and YGGFL, respectively). Therefore, the pentapeptide conformation affects the stability of the pentapeptide/β-CD inclusion complex. In contrast, the pentapeptide/α-CD inclusion complex was not affected by the oligopeptide conformation (27, 20, and 20 M^−1^ for free Try, YIGSR, and YGGFL, respectively).

The same year, Lovatt and co-workers investigated the dissociation of bovine insulin oligomers induced by aqueous solution of α-, HP-β-, and ME-β-CD [89]. The energetics of the dissociation of insulin oligomers have been investigated by microcalorimetry. As expected, the dissociation of insulin oligomers is increased upon the addition of CDs. This dissociation is clearly related to the interaction of these CDs with the protein side chains. Indeed, the dissociation of insulin oligomers is endothermic without CDs whereas in the presence of α-CD, the dissociation is less endothermic due to the exothermic binding of α-CD to exposed groups on insulin monomers after dissociation. Therefore, the α-CD facilitates the oligomer dissociation. In contrast, the dissociation is observed to be more endothermic in the presence of HP-β- and ME-β-CD although oligomer dissociation is induced. The authors suggest that the binding of HP-β- and ME-β-CD is endothermic and entropy driven.

As native and modified CDs are able to complex some amino acids which constitute peptides and proteins, these molecules can be useful for their separation by capillary zone electrophoresis. In this context, Rathore and Horváth reported that carboxymethyl-β-CD (CM-β-CD) in the electrophoretic medium (aqueous buffer solution, pH 2.5) enhanced the separation in capillary zone electrophoresis (raw fused-silica) of standard proteins such as α-chymotrypsinogen A, cytochrome *c*, lysozyme and ribonuclease A [90]. The obtained results proved that the separation of peptides and proteins can be enhanced by adding CDs to the electrophoretic medium. Unfortunately, only certain CDs can be used as selectivity enhancers. Indeed, in contrast to CM-β-CD, the addition of DM-β-CD had no effect on the separation of the mentioned proteins and peptides.

**Scheme 3:**
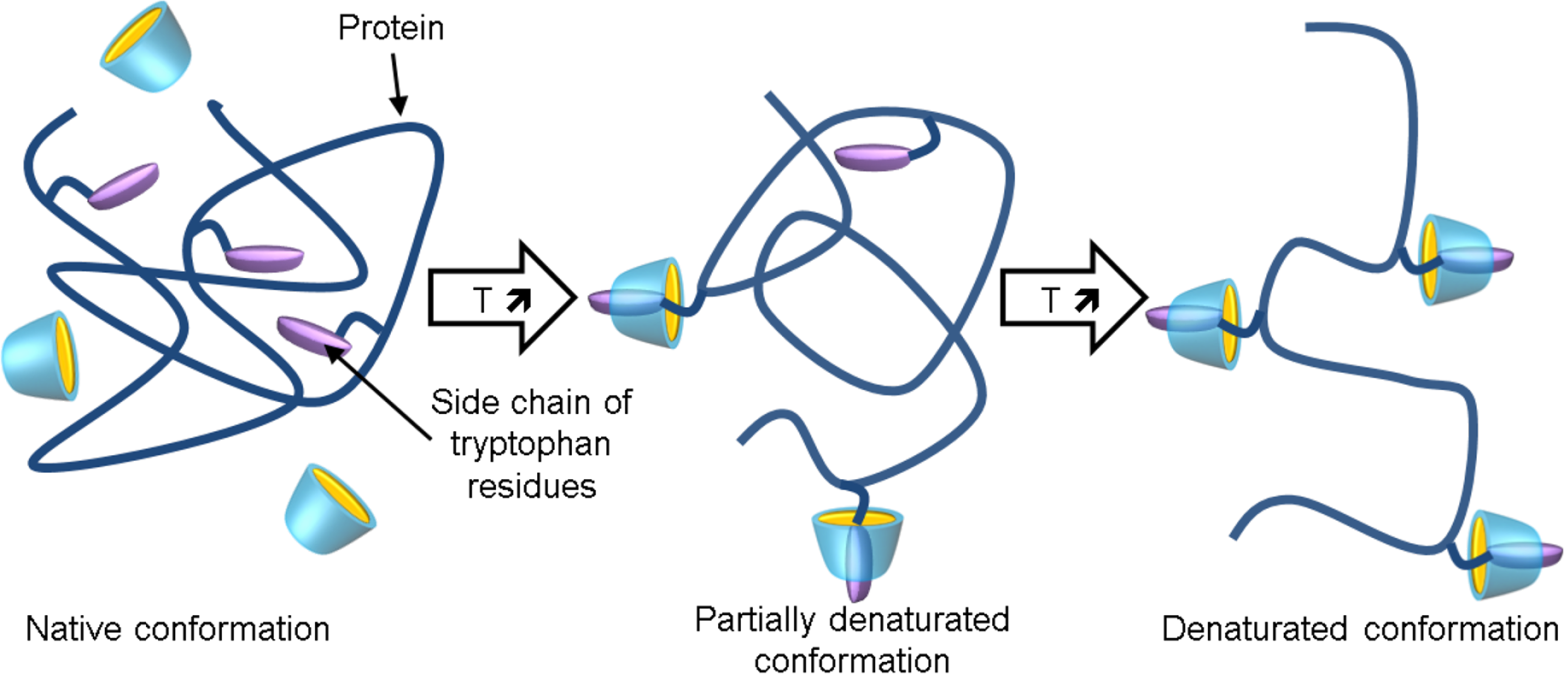
Proposed mechanism for the conformational change of egg white lysozyme with temperature elevating in the presence of modified CDs.

In 2006, the group of Yamamoto studied the effect of β-, γ-, G1-β-, and Me-α-CD on the thermal stability of an aqueous buffered solution of chicken egg white lysozyme by circular dichroism and fluorescence spectroscopy [91]. The thermal stability is significantly lowered in the presence of β-, γ-, and G1-β-CD whereas the opposite is true for the Me-α-CD. It should be noted that the thermal stability reduction is very important for G1-β-CD. Based on fluorescence spectroscopy, the authors suggested that CDs include the side chains of tryptophan (Trp) residues of lysozyme within their internal cavities to diminish the hydrophobicity of the hydrophobic core of lysozyme and consequently to lower the thermal stability (Scheme 3). In addition, some CD molecules persist in binding to the side chains of Trp residues to retard the renaturation of lysozyme.

From the findings described above, it can be presumed that the effect of CD is directly linked to its ability to complex Trp and the behavior of Me-α-CD can be related to its cavity size. The binding constants are always weaker with modified α-CD than with functionalized β-CD (see discussion above). In 2009, a ^1^H NMR spectroscopic study revealed that the ^1^H NMR signals corresponding to Trp residues were shifted upon the addition of G1-β-CD due to encapsulation of the tryptophan residues in the G1-β-CD cavity [92]. In addition, the ^1^H NMR signals for cysteine 64 and isoleucine 98 were also influenced to a considerable extent with the addition of G1-β-CD. This allows the conclusion that these hydrophobic amino acid residues are also included by this CD. These results are highly compatible with the very important thermal stability reduction observed in the presence of G1-β-CD. Therefore, the interaction of CDs with proteins is very complicated due to the presence of many binding sites.

Thaumatins refer to a family of proteins present in the sweetness of the katemfe fruit (*Thaumatococcus daniellii* Bennett) endemic in West Africa. It is used worldwide in human nutrition and pharmacology as a sweetener, flavor enhancer or to mask bitterness and it is 100,000 times sweeter than sucrose. Thaumatin has been shown to bind to G-protein-coupled receptors (GPCRs) which are transmembrane proteins, responsible for signal transduction. Therefore, the interaction of CDs with thaumatin could be used to modify the interaction of thaumatin with GPCRs and to modify its sweet-taste profile. In this context, Thordarson et al. studied the interaction of α-CD with thaumatin [93]. The 1D and 2D NMR experiments revealed that α-CD binds to aromatic residues of thaumatin with a binding constant of 8.5 M^−1^. As the active binding site of the thaumatin protein is known, the authors have synthesized a heptapeptide (Lys-Thr-Gly-Asp-Arg-Gly-Phe) that mimics this binding site of thaumatin. The results show that α-CD binds to the C-terminal solvent accessible phenylalanine residue with a binding constant of 8.8 M^−1^. As the α-CD may interact with the active binding site on thaumatin, the regulation of the interaction of thaumatin with GPCRs is probably possible.

Varca et al. published on the possible applications in the formulations of protein-like structures, such as enzymes, peptides and amino acids, for pharmaceutical applications [94]. The authors highlight that the formation of cyclodextrin/protein supramolecular complexes can be used to improve their stabilizations. However, the intrinsic characteristics of guest proteins can be also modified. In addition, it is exceedingly clear throughout this paragraph that peptides and proteins have moderate binding constants with CDs compared to lipids.

#### iii) Complexation of carbohydrates

The International Union of Pure and Applied Chemistry (IUPAC) defines carbohydrates as a class of organic compounds containing a carbonyl group (aldehyde or ketone) and at least two hydroxy residues (OH). It is noteworthy that substances derived from monosaccharides by reduction of the carbonyl group, by oxidation of at least one functional group at the end of the chain in carboxylic acid, by replacement of one or more hydroxy groups by a hydrogen atom, an amine, a thiol or any similar group are also called carbohydrates. Carbohydrates are, with proteins and lipids, essential constituents of living organisms because they are key biological intermediates for energy storage. In autotrophs, such as plants, sugars are converted into starch whereas for heterotrophic organisms, such as animals, they are stored as glycogen. However, polysaccharides serve also as structural components: cellulose for plants and chitin for arthropods. Moreover, saccharides and their derivatives play key roles in the immune system, fertilization, blood clotting, information transfer, etc. For instance, the 5-carbon monosaccharide ribose forms the backbone of the genetic molecule RNA (see below) and is also an important component of coenzymes (ATP, FAD and NAD).

In 1992, Aoyama et al. reported on the selective complexation of pentoses and hexoses by β-CD [95]. Based on competitive inhibition of the 8-anilinonaphthalene-1-sulfonate binding followed by fluorescence measurements, the binding constants can be estimated. The obtained results reveal that D-ribose, D- and L-arabinose, D-xylose, D-lyxose, D-2-deoxyribose, and methyl β-D-ribopyranoside were complexed by β-CD (binding constants ≤14 M^−1^). In contrast, aldohexoses and their derivatives (D-glucose, D-galactose, D-mannose, D- and L-fucose, and methyl α-D-fucopyranoside) were not complexed (binding constants ≈ “0” M^−1^). These binding constants can be directly correlated to the hydrophobicity of the sugar. Nevertheless, the H-bonds between the hydroxy groups of bound sugar and the OH groups of β-CD are also extremely important for determining the structure and for the selectivity of the complex.

It is noteworthy that several other publications have studied the interaction of D-glucose with native CDs. For instance, Hirsh and co-workers estimated the binding constants of D-glucose to α-CD and β-CD at 450 and 420 M^−1^, respectively, from blood glucose meter [96]. In contrast, Hacket et al. determined the binding constant of D-glucose to β-CD at 0.6 M^−1^ by fluorimetric competition titrations [97]. The results obtained by Hacket et al. are closer to the values published by Aoyama and co-workers. In addition, it is quite logical that D-glucose interacts weakly with native CDs due to its size and its hydrophilic property. However, from these conflicting results, Turner proposed to use kinetic measurements to determine the association constants of several sugars with β-CD [98]. This new method has been published in 2000 [99] and the binding constants obtained by these three groups are reported in Table 4.

**Table 4:** Association constants for the sugar/β-CD complexes (*K*).

	Guest	*K* (M^−1^)		

Pentoses	D-arabinose	0.7^a^	1.5^b^	0.9^c^
	D-ribose	5.3^a^	6.3^b^	4.8^c^
	D-xylose	1.0^a^	1.6^b^	1.6^c^
Hexoses	D-galactose	“0”^a^	0.5^b^	0.3^c^
	D-glucose	“0”^a^	0.6^b^	0.4^c^
	D-mannose	“0”^a^	1.0^b^	0.7^c^

^a^Taken from [95]. ^b^Taken from [97]. ^c^Taken from [98].

**Scheme 4:**
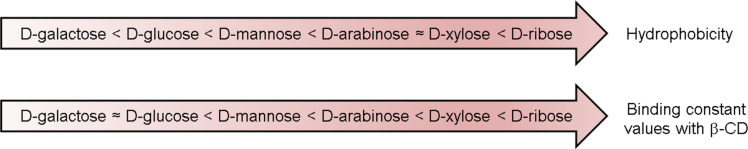
Sugar hydrophobicity scale according to Janado and Yano and correlation with the binding constant values observed with β-CD.

These values are relatively close to each other and the sugar/β-CD binding constants increase in the order of D-galactose ≈ D-glucose < D-mannose < D-arabinose < D-xylose < D-ribose. This magnitude is consistent with the order of magnitude of the sugar hydrophobicity scale determined by Janado and Yano in 1985 (Scheme 4) [100]. This hydrophobicity scale is corroborated by Wei and Pohorille for the hexose series [101]. Therefore, even if all the literature values for the binding constants obtained by the different methods are not especially self-consistent, it is clear that β-CD can selectively recognize pentoses in contrast to hexoses [102]. However, the binding constants remain very small (see Table 4). Based on all these results, the interaction of CDs with carbohydrates in aqueous solution can be completely neglected. Similar conclusions were made by Paal and Szeijtli [103].

#### iv) Complexation of nucleic acids

Nucleic acids are macromolecules, where the monomer is the nucleotide. Each nucleotide has three components: a 5-carbon sugar, a phosphate group, and a nitrogenous base. These nucleotides are joined by phosphodiester bonds. There are two types of nucleic acids according to the sugar: deoxyribose and ribose for deoxyribonucleic acid, DNA, and ribonucleic acid, RNA. Nucleic acids function in encoding, transmitting and expressing genetic information. As nucleic acids allow the synthesis of proteins their modifications result in numerous consequences. As earlier mentioned, CDs are used for numerous commercial applications. Therefore, the investigation of nucleic acid interactions (e.g., DNA or RNA) with various types of CDs is important to evaluate possible intracellular effects of CDs.

The interactions between native CDs and nucleic acids are still a subject of intense discussion along the past years. For instance, the results found in the literature for the α-CD are contradictory. Indeed, the works of Komiyama [104], Tee [105], and Spies et al. [106] suggested that α-CD cannot interact with DNA because the cavity of this molecule is too small to accommodate DNA base pairs. All these results support the work of Hoffmann and Bock who examined the complex formation between different CDs and nucleotides [107]. In contrast, in a more recent work, Jaffer et al. have found that α-CD can form H-bonds with DNA base pairs that flip out spontaneously at room temperature leading to DNA denaturation [108]. Consequently, exclusion and inclusion complexes are achieved with α- and β-CD, respectively. Nevertheless, it is noteworthy that when a complex is formed with β-CD, the ribose and phosphate groups of the nucleotides exert also a stabilizing effect by establishing H-bonds with the outer rim of the CD molecules. Interestingly, the extent of complexation depends significantly on the base composition and the double- or triple-helical structures. In contrast to native CDs, cationic CDs are known to interact strongly with DNA [109–110]. As consequence, CDs can be used to complex DNA and to encapsulate it into liposomes for potential gene therapy applications [111]. However, other formulation can be used to obtain non-viral vectors [112].

**Scheme 5:**
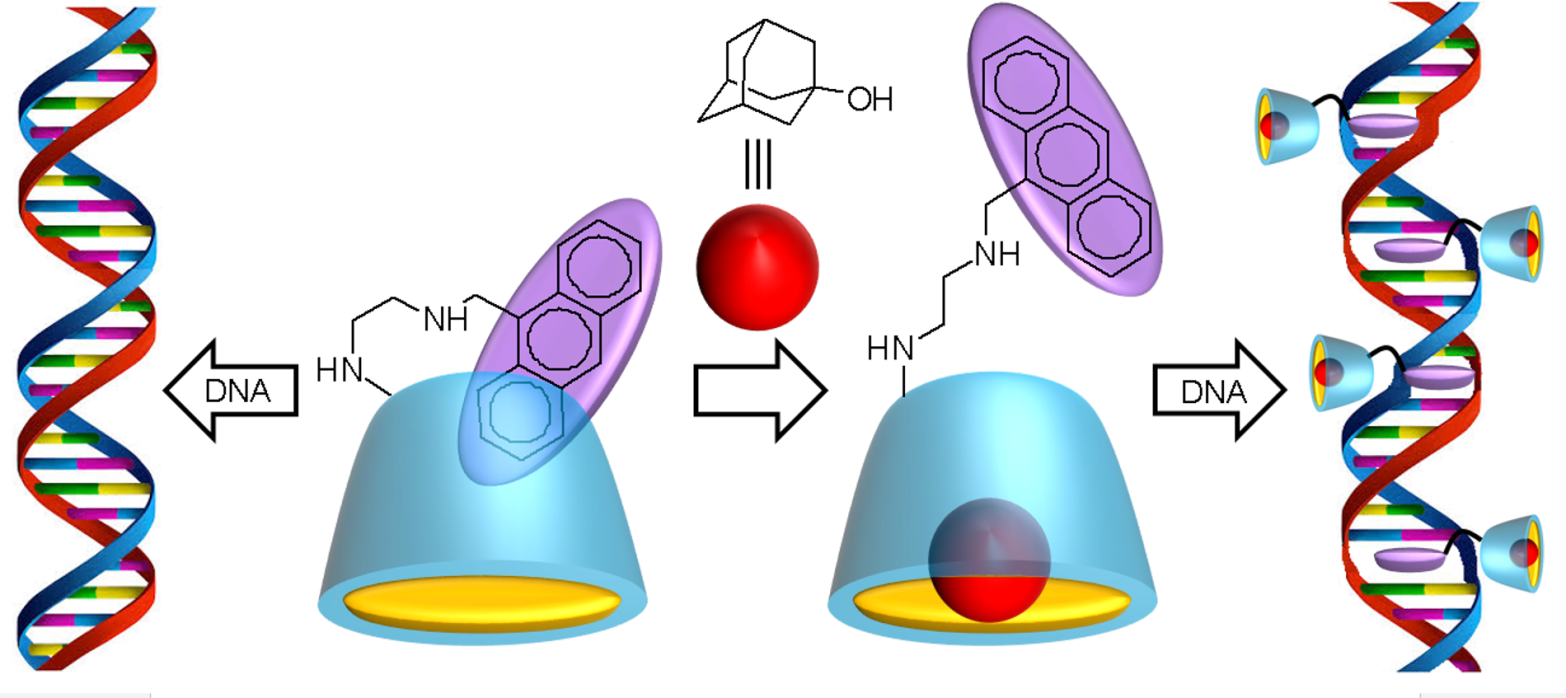
Principle of chemically switched DNA intercalators based on anthryl(alkylamino)-β-CD/1-adamantanol (Left: unchanged DNA strand. Right: DNA strand intercalated at four locations).

Since anthrylamines have potent DNA-intercalating properties, Ikeda et al. have attached an anthrylamine to a β-CD [113]. The obtained anthryl(alkylamino)-β-CD was used as chemically switched DNA intercalator. However, as the anthryl residue is locked in the CD cavity, its intercalation into DNA is not possible in aqueous solution. Upon addition of a ligand that is tightly bound in the CD cavity (e.g., 1-adamantanol), the host molecule releases the anthryl unit, which then leads to strong intercalation with the double-stranded DNA molecule leading to structural distortions (Scheme 5). This behavior was clearly established from ^1^H NMR spectroscopy (shifts and broadening of anthryl signals) in the presence of the 1-adamantanol guest. This concept could be very useful in nucleic acid reactions of medicinal and biotechnological importance for new drug delivery systems. Unfortunately, the binding constants between CDs and nucleic acids remain relatively modest and close to those observed for peptides and proteins (see above).

### Current and potential medical and biological applications

As mentioned earlier, CDs are able to complex biomolecules. Unfortunately, the strength of this behavior depends of the molecular structure. For instance, the binding constants increased in the order carbohydrates << nucleic acids << proteins < lipids. Consequently, the majority of biological investigations about CDs involved their ability to extract lipids (cholesterol or phospholipids) from the plasma membrane. As expected, this capacity can be very useful for numerous applications. For sake of clarity, only some typical applications of CD/cellular interactions are reported.

#### i) Cell membrane cholesterol efflux

As previously mentioned, CDs are able to interact and to complex cholesterol and others lipids [114]. A great number of publications deals with this topic and with the consequences of this phenomenon (e.g., hemolysis or cytotoxicity, see section above). Since the nineties, β-CDs are known to have a high affinity, in vitro, for sterols as compared to other lipids [58,115]. Consequently, these molecules can be used to manipulate the cellular cholesterol content, to modify cholesterol metabolism [115–116] and to stimulate the removal of cholesterol from a variety of cells in culture [80,117–119]. It should be noted that the cholesterol extraction by CDs is both time and dose dependent. In addition, the exposure of cells to modified β-CD in the 10–100 mM concentration range results in high rates of cell cholesterol efflux. Some typical examples are presented in this section.

CDs have been used to demonstrate the presence of different kinetic pools of cholesterol within cell models. Indeed, CDs have been used recently to monitor the movement of cholesterol from monolayers [57] or liposome bilayers [60]. For instance, a typical paper has been published in 2001 by Leventis and Silvius [60]. In order to characterize the CDs capacity to bind cholesterol, the authors examined the catalytic transfer of cholesterol between liposomes composed of 1-stearoyl-2-oleoyl phosphatidylcholine (SOPC) or SOPC/cholesterol. In the steady state under such conditions where a negligible fraction of the sterol is bound to CD (i.e., in the presence of submillimolar concentrations), β- and γ-CDs accelerate considerably the rate of cholesterol transfer between lipid vesicles (63- and 64-fold, respectively). This improvement is clearly greater than the transfer of phospholipid. The opposite is true for α- and methyl-β-CD. The kinetics of CD-mediated cholesterol transfer indicates that the transbilayer flip-flop of cholesterol is very rapid (halftime < 1–2 min at 37 °C). In the case of β-CD, the author reported on the relative affinities of cholesterol for different phospholipids. As expected, strong variations in cholesterol affinity were observed depending on the degree of chain unsaturation and the headgroup structure. The transfer revealed that cholesterol interacts with markedly higher affinity with sphingolipids than with other membrane phospholipids. As extension of this work, Huang and London highlighted the possibility of preparing asymmetric vesicles during the exchange of membrane lipids between different vesicles by selective inclusion of phospholipids and/or cholesterol into the CD cavity [120]. Moreover, CDs can also be used to monitor the intracellular movement of cholesterol in tissue culture cells [121].

As the cholesterol extraction by CDs occurs usually at very high rates, CDs have been used to demonstrate the presence of different kinetic pools of cholesterol within cells. Unfortunately, only few papers have studied the dynamics of this process on cells. For instance, the kinetics of cholesterol efflux have been examined in different cell lines such as fibroblasts [117], human erythrocytes [122], rat cerebellar neurons [123], differentiated human neurons and astrocytes [124], etc. All these results indicated that CDs induce cholesterol, sphingolipids, and phospholipids extraction from the cytoplasmic membrane typically in a range of 50–90% of the original amount. Castagne and co-workers studied the cholesterol extraction of native and modified β-CDs on endothelial cells (HUVEC) [125]. The measurement of the residual cholesterol content of cells reveals that cholesterol was extracted in a dose dependent relationship. As expected, a correlation was obtained between the cytotoxicity and the affinity for cholesterol. The affinity of CDs for cholesterol was classified in the order β-CD < HP-β-CD < Me-β-CD. Similar results are obtained with other biological membranes [117–126]. Another typical example has been published by Steck et al. The authors investigated the cholesterol movement created by the treatment of human erythrocytes with Me-β-CD [122]. The results show that the rate of efflux is approximately three orders of magnitude higher than the cholesterol transfer from cells to synthetic vesicles. Therefore, Me-β-CDs are very efficient to extract large amounts of membrane cholesterol at a very high rate. CDs can also catalyze the exchange of cholesterol between serum lipoproteins and cells [56].

#### ii) Cardiovascular diseases

**Scheme 6:**
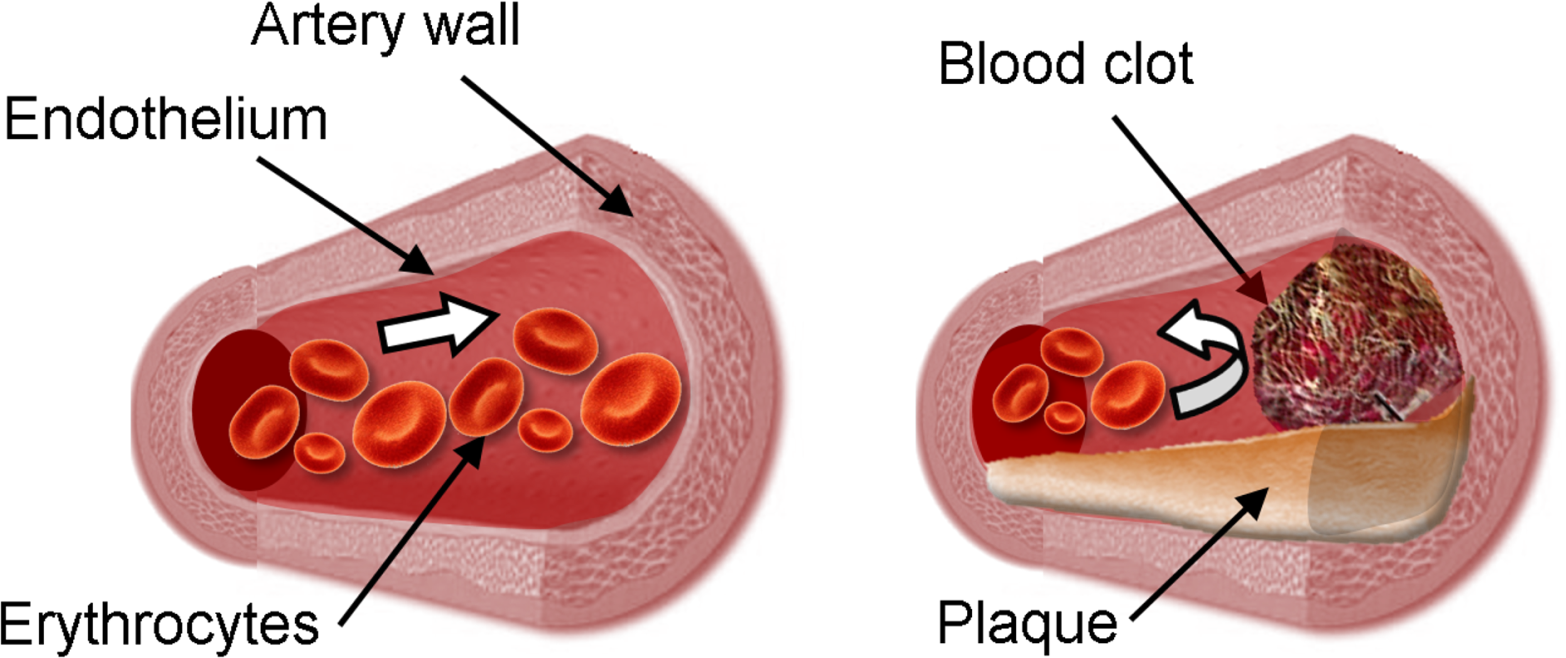
Normal (left) and diseased artery (right).

The atherosclerosis vascular disease (ASVD) is caused by an inflammation of the arterial wall that is caused by increased cholesterol blood levels and an accumulation of cholesterol crystals in the subendothelial spaces leading to arteriosclerotic plaque formation [127]. It is noteworthy that the cholesterol represents a maximum of 10% of the total mass of plaque. Consequently, the elasticity of the artery walls is reduced, pulse pressure can be modified and blood clot can be formed (Scheme 6). Cardiovascular disease is currently the leading cause of death worldwide. As plasma levels of cholesterol are associated with cardiovascular morbidity and mortality, the use of CDs to solubilize and to remove cholesterol (and plaque) is very promising to combat this deadly condition.

It is noteworthy that high concentrations of modified β-CDs result in rates of cell cholesterol efflux far in excess of those achieved with physiological cholesterol acceptors such as high-density lipoproteins (HDL). Indeed, plasma levels of HDL are inversely associated with cardiovascular morbidity and mortality because this lipoprotein is responsible for transporting cholesterol to the liver where it can be eliminated [128]. The opposite holds for low-density lipoproteins (LDL). Their function is to transport cholesterol, free or esterified, in the blood and through the body to bring them to the cells. HDL particles also reduce macrophage accumulation, and thus help prevent or even regress atherosclerosis. The alteration of cellular cholesterol regulation, named the reverse cholesterol transport, RCT, could be used to block atheroprogression associated with different severity degrees of atherosclerosis pathogenesis. From the pioneering works of Irie et al., it became clear that CDs can be useful to prevent atherosclerosis [115,129].

As the critical step in the formation of atherosclerosis plaque is the recruitment of monocytes (a type of white blood cells), which can differentiate into macrophages and ingest LDL, Murphy et al. proposed to prevent the activation/expression of monocyte adhesion [130]. For this cell adhesion, molecules such as CD11b are required. Therefore, the authors reported that β-CD, but not its cholesterol complex, inhibits CD11b activation. As the cholesterol content of lipid rafts diminished after treatment with the cholesterol acceptors, the authors proposed that the cholesterol efflux from serum monocytes is the main mechanism and is probably an effective means of inhibiting the development of atherosclerotic plaques.

In 2015, Montecucco et al. reported the anti-atherosclerotic action of KLEPTOSE^®^ CRYSMEB (a mixture of methylated β-CD where 2-*O*-methylations are dominant) in atherosclerotic mouse models [131]. As expected, their interfering action with cholesterol metabolism has a positive impact on atherogenesis, lipid profile and atherosclerotic plaque inflammation. In addition to reduce triglyceride serum levels, this CD reduces cholesterol accumulation in atherosclerotic plaques by the modification of HDL-cholesterol levels. It is noteworthy that HDL and apolipoprotein A-I (ApoA-I) cause a dose-dependent reduction in the activation of CD11b (i.e., anti-inflammatory effect on monocytes) through interactions with several receptors and ABCA1 for HDL and ApoA-I, respectively.

However, the process, which leads to an aberrant accumulation of cholesterol in artery walls forming atherosclerotic plaques, is complex. Thus the alteration of RCT as well as the expression and the functionality of transporters (ABCA1, ABCG1, and SR-BI) involved in this process could be very useful in the fight against atherosclerosis pathogenesis. As pointed out by Coisne and co-workers, “*RCT alterations have been poorly studied at the arterial endothelial cell and smooth muscle cells levels*” [132]. Consequently, the authors investigated the effect of different methylated β-CDs on the RCT of arterial endothelial and smooth muscle cells. It should be noted that these two cell types express basal levels of ABCA1 and SR-BI whereas ABCG1 was solely found in arterial endothelial cells. The authors highlighted the correlation between the percentages of cholesterol extraction and the methylation degree of the CDs. This effect was clearly independent of the membrane composition. The expression levels of ABCA1 and ABCG1, as well as the cholesterol efflux to ApoA-I and HDL, were reduced due to cholesterol-methylated β-CD interaction. Consequently, the cellular cholesterol involved in atherosclerotic lesions is lowered and the expression of ABCA1 and ABCG1 transporters involved in RCT is clearly modulated.

In 2016, Zimmer et al. published on the effect of HP-β-CD in order to reduce atherosclerotic plaques [133]. The HP-β-CD can be used to dissolve cholesterol crystal (responsible for the complex inflammatory response) which can be excreted from the body in urine. Mice were fed with a cholesterol-rich diet for 12 weeks in order to promote fatty plaques in their blood vessels (i.e., to obtain atherosclerotic mice). After 8 weeks, they started the injection of HP-β-CD (2 injections by week). Over the remaining four weeks, the authors observed a plaque reduction in atherosclerotic mice that had consumed HP-β-CD compared with plaques in the blood vessels of untreated animals (≈46% reduction). From a mechanistic point of view, the researchers suspect that the CD boosts the activity of macrophages, enabling them to attack excess cholesterol without causing inflammation. Indeed, CD increases liver X receptor (LXR) involved in the antiatherosclerotic and anti-inflammatory effects as well as in the RCT improvement.

Moreover, α-CD can also be used to reduce LDL cholesterol and alters plasma fatty acid profile [134–135]. In 2016, a double blind, placebo-controlled clinical trial has been published on the effect of oral α-CD [136]. After 12 to 14 weeks, a daily 6 gram dose of α-CD allowed to reduce fasting plasma glucose levels (1.6%, *p* < 0.05) and insulin index (11%, *p* < 0.04) in 75 healthy men and women. In addition, the LDL cholesterol levels were reduced by 10% (*p* < 0.045) compared with placebo. This CD was well tolerated and no serious adverse events were reported. Only about 8% of patients treated with α-CD reported side effect such as minor gastrointestinal symptoms (3% for the placebo). Consequently, the use of α-CD, safe and well tolerated, showed a reduction in LDL cholesterol, and an improvement of fasting plasma glucose.

The ability of CDs to change the contractibility of arterial smooth muscles indicates that the cellular cholesterol level is an extremely important factor for the cardiovascular system. Continued research on this front could potentially lead to major advancement in the fight against heart disease.

#### iii) Neurologic diseases

Like in other body systems, the cells of the nervous system are also susceptible to cholesterol extraction mediated by CDs. In the present section, for sake of clarity, only the potential applications of CDs to fight the Alzheimer’s and Niemann–Pick type C diseases (AD and NPC, respectively) are reported.

AD is a chronic neurodegenerative disease which represents 60% to 70% of cases of dementia. This disease is characterized by the formation of amyloid plaques in the brain and is often associated to the cerebral accumulation of amyloidogenic peptides (Aβ42). This production is mediated by two neuronal enzymes (β- and γ-secretase) which can be inhibited by methylated β-CDs via cholesterol depletion [137]. Additionally, Yao and co-workers demonstrated that HP-β-CD reduces cell membrane cholesterol accumulation in N2a cells overexpressing Swedish mutant APP (SwN2a) [138]. Moreover, this CD dramatically lowered the levels of Aβ42 in cells as well as the amyloid plaque deposition by reduction of APP protein β cleavage and by up-regulation of the gene expression involved in cholesterol transport. In cell models, this CD also improved clearance mechanisms.

CDs also exert significant beneficial effects in NPC disease, which shares neuropathological features with AD. This disorder is characterized by an abnormal endosomal/lysosomal storage disease associated with genetic mutations in NPC1 and NPC2 genes coding for proteins involved in the intracellular cholesterol transport. Consequently, functions of the impaired proteins cause a progressive neurodegeneration as well as liver and lung diseases. As these two proteins act in tandem and promote the export of cholesterol from endosomes/lysosomes, CDs can bypass the functions of NPC1 and NPC2 and can trap and transport membrane-stored cholesterol from endosomes/lysosomes [139]. This ability of CDs to sequester and to transport cholesterol could potentially lead to major advancements in our ability to fight neurodegenerative diseases.

#### iv) Antipathogen activities

Cholesterol levels in the plasma membrane are extremely important in many parts of the viral infection process such as the entry and release of virions from the host cell as well as for the transport of various viral proteins. CDs have a clear antiviral activity against influenza virus [140], human immunodeficiency virus (HIV-1) [141], murine corona virus [142], poliovirus [143], human T cell leukemia virus (HTLV-1) [144], Newcastle virus [145–146], varicella-zoster [147], duck and human hepatitis B virus [148–149], bluetongue virus [150], etc. In these cases, the ability of CDs to decrease membrane cholesterol was proposed as antiviral mechanism. Nevertheless, the biological effects of the CDs can be classified according to their role: i) to impede the viral entry in the host cell, ii) to decrease the relative infectivity of the virions, iii) to decrease the observed viral titer, and iv) to disrupt the surface transport of influenza virus hemagglutinin. Few typical examples of the CD effect on the pathogenicity of several viruses are reported.

The HIV is a widely studied virus in terms of the effects of CDs. For instance, sulfated CDs are able to inhibit HIV infection [151–152]. In 1998, Leydet et al. demonstrated anti-HIV and anticytomegalovirus activity of several charged CD derivatives [153]. In 2008, Liao et al. reported that HP-β-CD exhibits also an anti-HIV activity based on cholesterol depletion [154]. However, the mechanism had not yet been determined. Since the membrane cholesterol [155] and lipid raft-based receptors [156] are strictly required for infectivity and HIV entry, CDs are excellent candidates for its use as a chemical barrier for AIDS prophylaxis.

Another common viral disease is caused by herpes simplex virus (HSV) leading to several distinct medical disorders including orofacial and genital herpes or encephalitis [157]. In this context, the anti-HSV properties of native CDs (α- and β-CD) have been estimated against HSV-1 and HSV-2 [158]. The antiviral properties were clearly dependent on the cavity size: α-CD exhibited no significant antiherpetic activity, while, under similar conditions, β-CD reduced both the cell-free and cell-associated virus more effectively than acyclovir (i.e., antiherpetic drug). Indeed, the results reveal an almost complete protection of Vero cells against acyclovir-sensitive and acyclovir-resistant strains of HSV. The ability of β-CD to impede virus replication is proposed as antiviral mechanism.

**Scheme 7:**
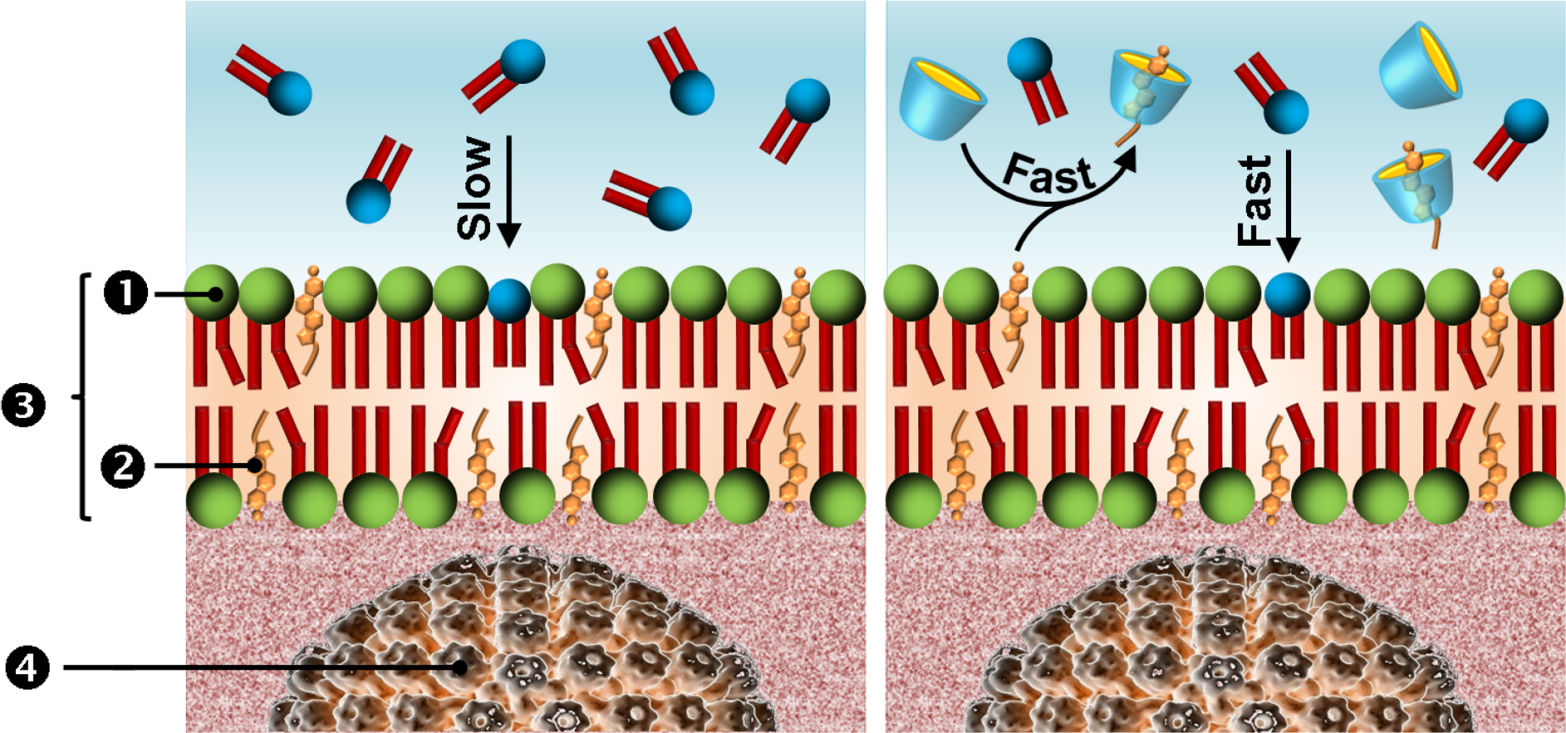
Kinetics of [DiC_10_] insertion into the viral envelope without (left) or with γ-CD (right). Note that, after this step, the presence of [DiC_10_] cations induces morphological changes that enhance the envelope fluidity and lead to the virus inactivation after envelope disruption (1. phospholipid, 2. cholesterol, 3. envelope, and 4. nucleocapsid).

The potential occurrence of synergistic effects presents a special case, and may occur when one substance increases the activity of another. Currently, gaps in our knowledge of the circumstances under which such effects may occur (e.g., mixture composition, contact time, species, and exposure concentrations) often hamper predictive approaches. However, since the CDs are able to extract cholesterol and other lipids from the viral membrane, it is likely that their combination with virucides or antiviral drugs which act on the same target results in a synergistic effect. Based on this assumption, our group studied the combination of di-*n*-decyldimethylammonium chloride, [DiC_10_][Cl] (the most widely used cationic surfactant with intrinsic virucidal activity), and native CDs (α-, β- and γ-CD) [159]. A marked synergism was observed with γ-CD against lipid-containing deoxyribonucleic and ribonucleic acid viruses (HSV-1, respiratory syncytial virus, RSV), and vaccinia viruses, VACV). Indeed, noticeable reductions of the [DiC_10_][Cl] concentration (i.e., active virucide) were obtained: 72, 40 and 85% against HSV-1, RSV and VACV, respectively. In all cases, submillimolar [DiC_10_][Cl] and γ-CD concentrations were required to obtain a “6-log reduction” (equivalent to 99.9999% reduction) of the viral titer. Therefore, for these diluted solutions, free CD and [DiC_10_] species prevail due to the Le Châtelier’s principle. Moreover, the micellization equilibrium is not relevant as the virucidal activity was clearly obtained in the premicellar region. Thus, the proposed mechanism of the synergy is based on the ability of CD to extract rapidly cholesterol from the viral envelope. Indeed, γ-CD catalyzes the rapid exchange of cholesterol between the viral envelope and the aqueous solution. The sequestration of cholesterol in the bulk phase facilitates the [DiC_10_] insertion within the lipid envelope which leads to the virus inactivation (Scheme 7). This means that γ-CD accelerates the rate of cholesterol extraction by a larger factor than α- or β-CD. The proposed mechanism is highly compatible with the results of Leventis and Silvius (see above) [60]. These results demonstrate a clear effect of CDs on the “viability” of enveloped viruses and provide evidences of their potential use in order to improve the efficiency of common antiviral medications.

As cholesterol extraction is general and not limited to viral infections, a whole range of studies have shown that the presence of CDs impedes the entry of bacteria, fungi and parasites into host cells. This effect has been demonstrated for *Plasmodium* species [160], *Campylobacter jejuni* [161], *Leishmania donovani* [162], etc. and this behavior can be explained by the vital role of the lipid rafts in the binding and the entry of pathogens into host cells. Therefore, synergistic effects can also be obtained for bacteria, fungi and parasites. For instance, the combination of [DiC_10_][Cl] and β-CD allows a clear reduction of the minimum inhibitory concentration, MIC, against *Candida albicans* compared to [DiC_10_][Cl] alone. This effect was only observed for the β-CD/[DiC_10_] mixture: the MIC values for α- and γ-CD/[DiC_10_] mixtures were similar to that of [DiC_10_][Cl] alone. This behavior was attributed to the interaction of β-CD with the lipid membrane components [163]. Other relevant examples can be found in the review of Macaev et al. [164].

## Conclusion

This review proposes an overview of the current and potential applications of CDs throughout their interactions with endogenous substances that originate from within an organism, tissue or cell. The majority of these applications are based on the capacity of CDs to withdraw cholesterol of the plasma membrane. This behavior presents several applications such as cholesterol manipulation, control of viral and bacterial infections, treatment of Alzheimer’s and heart diseases, etc. Moreover, CDs present a viable basis in the context of “green pharmacy and medicine”. In the last decade, the concept of “eco-friendly pharmacy” emerged in response to the Kreisberg’s question: “*what clinicians can do to reduce the environmental impacts of medications”* [165]? Of course, the answers are based on similar principles than green chemistry initially developed by Anastas and Warner [166]. The principles cover various concepts such as: i) the use of bio-sourced ingredients, ii) the use of “green concepts” during the production (chemicals, synthesis processes, life cycle engineering, packaging, waste management), iii) the reduction of the negative impact of medication transportations, iv) the reduction of healthcare environmental footprint, v) the reduction of the use of pharmaceuticals and, vi) the improvement of the ultimate drug disposal with the use of take-back programs [167]. As CDs are bio-sourced compounds with very low toxicity dangers and easily biodegradable, they can be used to obtain more sustainable drug formulations in which CDs act as an active green ingredient and not only as an excipient. It is noteworthy that these CDs can be used alone or in combination with common petro-sourced medications. If a synergistic effect between the two molecules is obtained, a significant amount of the drug can be replaced by eco- and biocompatible CDs whilst maintaining the same biological activity. This is particularly interesting as it solves at least partially the negative impact of pharmaceutical formulations to the environment. Consequently, in this context of “greener pharmacy”, CDs will contribute without doubt to preserve our planet in the coming years.
